# Medication-related problems identified by community pharmacists: a descriptive case study of two Australian populations

**DOI:** 10.1186/s40545-023-00637-x

**Published:** 2023-11-02

**Authors:** Jack C. Collins, Jie Hu, Sara S. McMillan, Claire L. O’Reilly, Sarira El-Den, Fiona Kelly, Jean Spinks, Toni Riley, Amanda J. Wheeler

**Affiliations:** 1 The University of Sydney School of Pharmacy, Faculty of Medicine and Health, The University of Sydney, Sydney, NSW Australia; 2 Menzies Health Institute Queensland, Griffith University, Building N70, Nathan Campus, Nathan, Brisbane, QLD 4111 Australia; 3 Centre for Mental Health, Griffith University, Brisbane, QLD Australia; 4 School of Pharmacy and Medical Sciences, Griffith Health, Griffith University, Gold Coast, QLD Australia; 5 Centre for Business and Economics of Health, The University of Queensland, Brisbane, QLD Australia; 6 Faculty of Medical and Health Sciences, University of Auckland, Auckland, New Zealand

**Keywords:** Medication review, Pharmacists, Drug therapy, Community pharmacy services, Mental illness, Australian Aboriginal and Torres Strait Islander Peoples

## Abstract

**Background:**

Medication-related problems (MRPs) contribute significantly to preventable patient harm and global healthcare expenditure. Vulnerable populations, including Indigenous Australians (please note that the use of the term ‘Indigenous’ in this paper includes all Aboriginal and Torres Strait Islander people and acknowledges their rich traditions and heterogenous cultures.) and people living with severe and persistent mental illness (SPMI), may be at increased risk of MRPs. Pharmacist-led medication reviews can identify MRPs for targeted action.

**Objective:**

To characterize MRPs identified and recommendations made by community pharmacists during medication reviews conducted with Indigenous Australians and people living with SPMI.

**Methods:**

Participants were recruited through two Australian trials testing the feasibility and/or effectiveness of novel community pharmacist-led interventions, the Indigenous Medication Review Service (*IMeRSe*) feasibility study (June 2018–July 2019) and Bridging the Gap between Physical and Mental Illness in Community Pharmacy (*PharMIbridge*) randomized controlled trial (September 2020–December 2021). Trained community pharmacists conducted medication reviews responsive to the cultural and health needs of participants. MRPs, MRP severity and pharmacist recommendations were documented and classified using an established classification system (DOCUMENT). MRP severity was assessed by pharmacists and an independent assessor. Data were analysed descriptively, and paired t-tests were used to compare severity ratings.

**Results:**

Pharmacists identified 795 MRPs with 411 participants across both trials (*n* = 255 *IMeRSe*, *n* = 156 *PharMIbridge*). Non-adherence to medication was the most common (*n* = 157, 25.1%) and second-most common (*n* = 25, 14.7%) MRP in *IMeRSe* and *PharMIbridge*, respectively. Undertreatment was the second-most common MRP in the sample of Indigenous Australians (*n* = 139, 22.2%), and reports of toxicity/adverse reactions were most common in people living with SPMI (*n* = 41, 24.1%). A change in pharmacotherapy was the most frequent recommendation made by pharmacists (40.2% and 55.0% in *IMeRSe* and *PharMIbridge*, respectively). Severity ratings varied, with the majority being ‘Mild’ or ‘Moderate’ in both groups. Significant differences were found in the severity rating assigned by trial pharmacists and the independent assessor.

**Conclusions:**

Community pharmacists identified a range of MRPs experienced by two at-risk populations, most commonly non-adherence and toxicity or adverse reactions, when conducting medication reviews and proposed diverse strategies to manage these, frequently recommending a change in pharmacotherapy. These findings highlight the opportunity for more targeted approaches to identifying and managing MRPs in primary care and tailored community pharmacist-led interventions may be of value in this space.

*Trail Registration*: Australian and New Zealand Clinical Trial Registry records (IMeRSe ACTRN12618000188235 registered 06/02/2018 & *PharMIbridge* ACTRN12620000577910 registered 18/05/2020).

**Supplementary Information:**

The online version contains supplementary material available at 10.1186/s40545-023-00637-x.

## Introduction

Medication (drug)-related problems (MRPs) contribute significantly to morbidity, hospitalization, and subsequent healthcare expenditure around the world [1]. MRPs have been defined as “an event or circumstance involving drug therapy that actually or potentially interferes with desired health outcomes” [2] and may occur at any stage of the medication-use process, from prescribing, to dispensing and use by consumers. Although definitions and classifications of MRPs vary, examples include adverse drug reactions, medication errors, and adverse drug events [3]. Factors such as polypharmacy, older age, or comorbid conditions contribute to risk of MRPs [4]. It is estimated that anywhere up to a median of 12.1% of all MRPs result in hospitalization [4] and contribute significantly to preventable patient harm [5]. In Australia alone, an estimated 250,000 hospital admissions annually are due to MRPs which costs the health care system (AUD) 1.4 billion dollars [6].

As medication experts, pharmacists have a critical role in the identification and management of MRPs [7]. Medication reviews, a structured evaluation of medications to optimize medication use and health outcomes [8], are an example of a pharmacist-led intervention with the intention of reducing the number and impact of MRPs experienced by consumers [8, 9]. Although outcomes of medication reviews have varied [10, 11], they form a routine part of community pharmacy practice in many countries [12–15]. In Australia, pharmacists can provide several government-funded medication reviews to eligible consumers. These include in-depth medication management reviews intended to be conducted in the consumer’s place of residence (Home Medicines Review and Residential Medication Management Review) and brief in-pharmacy medication reviews (Diabetes MedsCheck and MedsCheck) to eligible services [16–18].

Certain populations may also be at greater risk of experiencing MRPs which may result in negative health outcomes. For example, Indigenous Australians^1^ may be at increased risk of experiencing MRPs [19]. Multimorbid illnesses, poor health literacy and lack of access to culturally appropriate information sources [20, 21] may contribute to the risk of unidentified MRPs. Furthermore, Indigenous Australians report barriers to accessing existing medication review services [22] and these services may not meet the health and cultural needs of this group. Another at-risk and underserved population are people living with severe and persistent mental illness (SPMI),^2^ which includes conditions such as schizophrenia, bipolar disorder, and major depression [23]. People living with SPMI have been identified to have increased risk and high incidence rates of MRPs due to numerous reasons, including high prevalence of poorly managed physical illnesses and the adverse effects associated with psychotropic (e.g. antipsychotics) medication use [24–26]. Lack of training and confidence in caring for people with mental illness [27] as well as stigmatizing attitudes held by healthcare professionals towards this group [28] may generate barriers to access to, and provision of, existing medication review services.

In recognition of the health needs and access barriers experienced by these two populations, two novel community pharmacist-delivered interventions, which included medication review as a component of the intervention, were trialed in Australia. These interventions were the Indigenous Medication Review Service (*IMeRSe*), trialed in the *IMeRSe* feasibility study [29], and the Bridging the Gap between Physical and Mental Illness in Community Pharmacy (*PharMIbridge*) intervention, trialed in the *PharMIbridge* randomized controlled trial (RCT) [30].

Given the increased risk of MRPs and potentially unmet needs of these two populations, the primary aim of the current study was to examine these two populations as case studies to characterize the MRPs identified and subsequent recommendations made by community pharmacists during medication reviews (interventions) conducted as part of the *IMeRSe* feasibility study and the *PharMIbridge* RCT. A secondary aim was to examine the severity (clinical significance) rating of MRPs by trial pharmacists and an independent accredited pharmacist.

## Methods

Data presented in this paper were collected as part of the *IMeRSe* feasibility study and the *PharMIbridge* RCT. Further information on the eligibility criteria, interventions, and methods can be obtained from the respective published protocols [29, 30] and the Australian and New Zealand Clinical Trial Registry records (ACTRN12618000188235 and ACTRN12620000577910, respectively). Other relevant findings arising from both trials will be published elsewhere.

### Ethical approval

The *IMeRSe* feasibility study and *PharMIbridge* RCT received ethical approval from the Griffith University Human Research Ethics Committee (HREC/2018/251 and HREC/2019/473, respectively). Written informed consent was obtained from all consumer participants.

### Study design and setting

#### IMeRSe feasibility study

The *IMeRSe* feasibility study developed and evaluated a culturally responsive medication management service, which was delivered by community pharmacists in collaboration with Aboriginal Health Service (AHS) staff. During the trial, 291 Aboriginal and Torres Strait Islander participants aged 18 years and over, with chronic conditions and at-risk of MRPs (full eligibility criteria available elsewhere [29]), were recruited from nine Aboriginal Health Services across three Australian jurisdictions (Northern Territory, Queensland and New South Wales). Pharmacists from 23 community pharmacies associated with the AHSs and AHS staff completed online modules and attended a one-day face-to-face training workshop prior to delivering the intervention. Pharmacists were also supported by mentors throughout the trial [31]. Training included two case studies with recorded vignettes which guided pharmacists through the trial procedures, including a strengths-based approach to medication review. Data from 255 consumer participants who had completed the baseline questionnaire and the intervention (called a *Medicines Talk;* see “IMeRSe feasibility study”) with a trained community pharmacist, were analysed.

#### *PharMIbridge* RCT

The *PharMIbridge* RCT was conducted in 51 community pharmacies across four Australian regions (Australian Capital Territory, Hunter New England, Northern Sydney, and regional Victoria). Recruited community pharmacies were randomized into the intervention (IG) or comparator group. Consumers aged 16 years of age and over, living with a self-reported SPMI and taking at least one antipsychotic or mood-stabilizer medication were recruited from IG community pharmacies (full eligibility criteria available elsewhere [30]). Pharmacists and pharmacy support staff from IG pharmacies attended a two-day face-to-face training workshop prior to delivering the intervention [32]. The training included a case study with recorded vignettes about a consumer experiencing MRPs as well as a presentation on psychopharmacology and other relevant topics including physical health issues and motivational interviewing. This paper reports on IG data only, from 156 consumer participants who completed the baseline survey and received the intervention (called *Initial Health Review*; see “PharMIbridge RCT”) within 25 community pharmacies.

### Data collection

#### *IMeRSe* feasibility study

Data collection occurred from June 2018–July 2019. Consumer participants completed an electronic or paper baseline questionnaire with a study coordinator from the AHS and proceeded to the *Medicines Talk* during which the pharmacist reviewed current medications, identified potential MRPs and other issues of concern, and facilitated goal setting to improve their general health. The intervention was intended to be holistic and was based on the *Stay Strong* app [33], a resource culturally adapted for Indigenous people. An Aboriginal Health Worker (AHW) or other AHS staff coordinated the *Medicines Talk* and any follow-up services (at an interval determined by both parties). The *Medicines Talk* could include the participant, an AHW, and/or family members according to participant preference.

Demographic and health characteristics including age, gender, cultural identity, and chronic conditions were obtained. A research-specific module was developed in community pharmacy clinical practice software (GuildCare NG™) to collect and document the *Medicines Talk,* including information on current medications, MRPs and associated recommendations or pharmacist action/s. A Medicines Report was subsequently generated and made available to the nominated General Practitioner (GP) through the module for further review and action. Service coordination was conducted by the AHS staff member within this bespoke shared online module.

#### *PharMIbridge* RCT

Data collection occurred from September 2020–December 2021. Similar to *IMeRSe*, an electronic baseline survey was used to capture participant demographic details and responses to numerous validated instruments encompassing general health, medication adherence, mental wellbeing, physical wellbeing, and substance use, among others [30]. As part of the *Initial Health Review*, consumers worked with pharmacists to review key baseline survey results, identify any potentially relevant issues (including MRPs) and set corresponding goals to address these issues. Although the intervention was intended to be holistic, with medication review forming a component of the intervention, emphasis was placed on physical health and wellbeing. Further instruments were made available to pharmacists to help with issue identification, including the My Medicines and Me Questionnaire (M3Q) which can assist in the detection of side-effects from psychotropic medication use [34].

Following the *Initial Health Review*, consumer participants could meet with pharmacists for *Follow-Up* services (at an interval determined by both parties) with a *Final Health Review* scheduled 6 months after the *Initial Health Review*. New and previously unidentified MRPs were documented during the *Follow-Ups* and *Final Health Review*. Issues and associated recommendations could be shared with a nominated healthcare professional (usually a GP) following any service. Similar to *IMeRSe*, documentation occurred in a bespoke *PharMIbridge* research module built into GuildCare NG™.

### DOCUMENT framework and severity rating

When a MRP was identified, pharmacists in both trials were required to classify the MRP and the potential severity of the MRP using the validated Australian DOCUMENT framework [35]. Although other classification systems exist, including the Pharmaceutical Care Network Europe (PCNE) system [2], DOCUMENT (which was informed by the PCNE system [35]) was selected as it was designed in Australia and used in a previously funded Australian professional pharmacy service [36, 37]. The DOCUMENT framework broadly classifies MRPs into eight categories: ‘Drug selection’, ‘Over or underdose’, ‘Compliance’, ‘Undertreated’, ‘Monitoring’, ‘Education or information’, ‘Not classifiable’, and ‘Toxicity or adverse reaction’. All categories except ‘Not classifiable’, have further sub-classifications. Severity of the potential impact of the MRPs if not resolved (i.e. clinical significance) was classified using five categories, ranging from ‘Nil’ to ‘High’, coded from 0 to 4, respectively [35]. Full descriptors of each of the severity classifications are available in Additional file 1: Table S1. Free-text fields were available for pharmacists to describe MRPs and associated recommendations. In *IMeRSe*, free-text recommendations were coded into an adapted version of DOCUMENT by research team members, whereas *PharMIbridge* pharmacists were asked to self-select the appropriate recommendation codes using DOCUMENT. The adapted version used in *IMeRSe* included an additional category of ‘Lifestyle recommendation’ which was not included in the categories in *PharMIbridge*. The severity rating of MRPs was also not documented for a proportion of the MRPs (*n* = 271, 43.4%) in the *IMeRSe* feasibility study as the feature was not functional in the *Medicines Talk* module until February 2019.

### Independent coding of severity rating

The severity of MRPs identified in *IMeRSe* and *PharMIbridge* were independently coded by one author who is an accredited consultant pharmacist in Australia with significant experience conducting medication reviews. The independent coder used notes and medication lists created by pharmacists during the medication review and other clinical documentation such as shared health summaries (where available) to independently assign a severity classification to each of the documented MRPs.

### Data cleaning and analysis

Prior to analysis, data were exported to Microsoft Excel^®^ (Microsoft Corp, Redmond, USA) for review. Data were screened by a member of the research team to ensure unrelated issues had not been incorrectly coded as MRPs by pharmacists. Where it was clear that an issue was not an MRP, such as those relating to exercise or interpersonal relationships, it was excluded from the analysis. A secondary sample (10%) was screened by another author as a quality check. Descriptive analyses were performed on participant characteristics and details of the identified MRPs including classifications, severity ratings as well as pharmacists’ recommendations. Results are presented separately for the two trial samples. Mean (standard deviation [SD]) and median (interquartile range [IQR]) were reported for continuous variables, whereas number (percentage) was reported for categorical variables. To compare the mean severity ratings from the community pharmacists and the independent coder, paired t-tests were used. The significance level was set at *p*-value less than 0.05 (two-sided).

## Results

A total of 411 consumer participants (*n* = 255 *IMeRSe*, *n* = 156 *PharMIbridge*) were screened for MRPs by trained community pharmacists, with 795 MRPs identified and classified using DOCUMENT. The large majority (*n* = 625) of these MRPs were identified in the *IMeRSe* feasibility study (rate of 2.45 MRPs per consumer participant) as compared to 170 MRPs identified in the *PharMIbridge* RCT (rate of 1.09 MRPs per consumer participant). Non-MRPs made up the remaining 137 and 474 issues documented in *IMeRSe* and *PharMIbridge*, respectively and were not included in the analysis presented in this paper. No discrepancies were found between coders in the 10% quality check.

### Participant characteristics

Consumer participant characteristics are outlined in Table 1. Compared to the *PharMIbridge* population, the consumer participants in *IMeRSe* were older (average age of 60.0 years vs. 48.1 years), more likely to identify as female (62.8% vs. 55.2%), more likely to reside in a rural/remote/non-metropolitan region (76.9% vs. 44.2%) and used a greater number of medications (median 7 vs. 6). Consumer participants in *IMeRSe* lived with a median of six self-reported chronic conditions (including mental illness diagnoses), whereas *PharMIbridge* consumer participants reported living with a median of one chronic condition in addition to their mental illness(es).

**Table 1:** Participant characteristics (*n* = 411)

Characteristics	*IMeRSe*^a^ (*n* = 255) *n* (%)	*PharMIbridge*^b^ (*n* = 156) *n* (%)
Age, years, mean (SD)	60.0 (11.7)	48.1 (12.6)
Age group in years		
< 45	21 (8.2)	62 (39.7)
45–54	58 (22.8)	44 (28.2)
55–64	83 (32.6)	37 (23.7)
65–74	66 (25.9)	10 (6.4)
≥ 75	27 (10.6)	3 (1.9)
Gender		
Male	95 (37.3)	69 (44.2)
Female	160 (62.8)	85 (54.5)
Not specified	–	2 (1.3)
Cultural identity		
Aboriginal	236 (92.5)	8 (5.1)
Torres Strait Islander	2 (0.8)	–
Both	5 (2.0)	–
Neither	–	137 (87.8)
Not specified	12 (4.7)	11 (7.1)
Location^c^		
Urban/metropolitan	59 (23.1)	87 (55.8)
Rural/remote/non-metropolitan	196 (76.9)	69 (44.2)
Self-reported general health		
Excellent	10 (4.1)	3 (1.9)
Very good	44 (18.1)	27 (17.4)
Good	112 (46.1)	66 (42.6)
Fair	59 (24.3)	40 (25.8)
Poor	18 (7.4)	19 (12.3)
Number of self-reported chronic conditions, median (IQR)	6 (4)	1 (3)^d^
Number of medications, median (IQR)	7 (6)	6 (4)

^a^Number of participants who received a *Medicines Talk* service

^b^Number of participants who received a *PharMIbridge* initial intervention service (*Initial Health Review*)

^c^In the *IMeRSe* feasibility study, location was classified using the Pharmacy Accessibility and Remoteness Index of Australia 2018–2019 [50]; in the *PharMIbridge* RCT, location was classified using the Modified Monash Model [51]

^d^Excludes mental illnesses

### Classification of MRPs

Classification of the 795 MRPs identified in *IMeRSe* and *PharMIbridge* is shown in Table 2. Of the 625 MRPs experienced by 255 Indigenous Australians in *IMeRSe*, pharmacists commonly classified MRPs as ‘Compliance’ (Adherence) (25.1%) followed by ‘Undertreated’ (22.2%), with ‘Toxicity or adverse reaction’ being the least common (5.1%). In contrast, of the 170 MRPs identified from 156 people living with SPMI in *PharMIbridge*, pharmacists most frequently classified the MRPs as ‘Toxicity or adverse reaction’ (24.1%) followed by ‘Compliance’ (14.7%), with ‘Education or information’ being the least common (8.2%). Examples of MRPs from each of the DOCUMENT categories from both trials can be found in Additional file 1: Table S2.

**Table 2:** Classification of MRPs (*n* = 795) by community pharmacists

Classification	Sub-classification	*IMeRSe* (total *n* = 625^#^) *n*	*PharMIbridge* (total *n* = 170) *n*
Drug selection	D0 Other drug selection problem	41	18
	D1 Duplication	7	0
	D2 Drug interaction	12	0
	D3 Wrong drug	2	2
	D4 Incorrect strength	1	1
	D5 Inappropriate dosage form	3	0
	D6 Contraindications apparent	8	2
	D7 No indication apparent	8	1
	Subtotal *n* (%)	81 (13.1)	24 (14.1)
Over or underdose	O0 Other dose problem	21	4
	O1 Prescribed dose too high	12	7
	O2 Prescribed dose too low	11	3
	O3 Incorrect or unclear dosing instructions	6	1
	Subtotal *n* (%)	50 (8.0)	15 (8.8)
Compliance	C0 Other compliance problem	47	6
	C1 Under-use by consumer	60	12
	C2 Over-use by consumer	5	1
	C3 Erratic use of medication	23	6
	C4 Intentional drug misuse (incl. over-the-counter medications)	3	0
	C5 Difficulty using dosage form	19	0
	Subtotal *n* (%)	157 (25.1)	25 (14.7)
Undertreated	U0 Other undertreated indication problem	19	1
	U1 Condition undertreated	65	13
	U2 Condition untreated	35	3
	U3 Preventative therapy required	20	1
	Subtotal *n* (%)	139 (22.2)	18 (10.6)
Monitoring	M0 Other monitoring problem	6	5
	M1 Laboratory monitoring	45	5
	M2 Non-laboratory monitoring	26	11
	Subtotal *n* (%)	77 (12.3)	21 (12.4)
Education or information	E0 Other education or information problem	36	7
	E1 Patient requests drug information	10	4
	E2 Patient requests disease management advice	6	3
	Subtotal *n* (%)	52 (8.3)	14 (8.2)
Not classifiable	N0 Clinical interventions that cannot be classified	36	12
	Subtotal *n* (%)	36 (5.8)	12 (7.1)
Toxicity or adverse reaction	T1 Toxicity, allergic reaction or adverse effect	32 (5.1)	41 (24.1)
	Subtotal *n* (%)	32 (5.1)	41 (24.1)

^#^Subtotal percentages do not total 100 due to rounding

### Severity of MRPs

Pharmacists in *IMeRSe* commonly rated the 354 MRPs that underwent severity classification (post-February 2019) as ‘Moderate’ (39.6%) or ‘High’ (28.0%). *PharMIbridge* pharmacists most frequently rated the severity of 170 MRPs as ‘Mild’ (47.7%) and ‘Moderate’ (32.9%), with ‘High’ less frequently applied (8.8%). The distribution of ratings across categories can be seen in Table 3. Examples of MRPs from each of the severity rantings from both trials can be found in Additional file 1: Table S3.

**Table 3:** Comparison of trial pharmacists and independent pharmacist rating of MRP severity

Potential severity rating	*IMeRSe* (*n* = 354*)		*PharMIbridge* (*n* = 170)	
	Pharmacist rated *n* (%)	Independent pharmacist rating *n* (%)	Pharmacist rated *n* (%)	Independent pharmacist rating *n* (%)
High (S4)	99 (28.0)	2 (0.6)	15 (8.8)	2 (1.2)
Moderate (S3)	140 (39.6)	171 (48.3)	56 (32.9)	113 (66.5)
Mild (S2)	79 (22.3)	56 (15.8)	81 (47.7)	46 (27.1)
Low (S1)	27 (7.6)	102 (28.8)	15 (8.8)	2 (1.2)
Nil (S0)	9 (2.5)	23 (6.5)	3 (1.8)	7 (4.1)
Mean score (SD)	2.8 (1.0)	2.1 (1.0)	2.4 (0.8)	2.6 (0.7)
P-value^#^	*p* < 0.001		*p* = 0.008	

*Rating of MRP severity was introduced into the GuildCare NG™ module during the course of the trial (Feb 2019); data only available for *Medicines Talks* that took place after this date is compared (*n* = 354). # Paired *t*-test

The independent consultant pharmacist reviewed pharmacist documented MRPs with pharmacist severity ratings (*n* = 354). The independent pharmacist coded the MRPs (0 = lowest severity rating and 4 = highest severity rating) as less severe than the community pharmacists in the *IMeRSe* sample, with a mean rating of 2.1 (SD 1.0) vs 2.8 (SD 1.0) (*t*(353) = 11.4, *p* < 0.001), and coded MRPs as more severe than the community pharmacists in the *PharMIbridge* sample, with a mean rating of 2.6 (SD 0.7) vs. 2.4 (SD 0.8) (*t*(169) = 2.7, *p* = 0.008) (Table 3).

### Pharmacist recommendations for identified MRPs

Pharmacists in both studies provided recommendation(s) to resolve or manage identified MRPs (see Tables 4, 5). A ‘Change in pharmacotherapy’ was the most common recommendation in both populations, making up 40.2% of 833 recommendations in *IMeRSe* and 55.0% of 278 in *PharMIbridge*, followed by ‘Monitoring’ in *IMeRSe* (19.3%) and ‘Referral to other services’ in *PharMIbridge* (27.7%). ‘No recommendation necessary’ was selected for three MRPs in *PharMIbridge*.

**Table 4:** Pharmacists’ recommendations (*n* = 833) for 625 MRPs documented in *IMeRSe* feasibility study

Recommendations/actions		*n*	%
Change in pharmacotherapy	Consider review of medication or dose	111	13.3
	Add new or additional medication	55	6.6
	Multiple recommendations	30	3.6
	Medication dose change	29	3.5
	Medication change	24	2.9
	Medication formulation change	21	2.5
	Dose frequency or schedule change	19	2.3
	Cease medication	18	2.2
	Recommend non-prescription medication^a^	16	1.9
	Recommend vaccination	8	1.0
	Medication brand change	4	0.5
	Subtotal	335	40.2
Referral to other services	Prescriber (GP)	52	6.2
	Other services, e.g. allied health	43	5.2
	Referral to multiple services	4	0.5
	Hospital	3	0.4
	Pharmacy-led services, e.g. weight loss	1	0.1
	Subtotal	103	12.4
Information provision	Education on medications or adherence	101	12.1
	Devices to enhance medication use, e.g. spacer	29	3.5
	Information related to multiple categories	13	1.6
	Recommendation to commence DAA	12	1.4
	Self-management plan, e.g. asthma action plan	8	1.0
	Subtotal	163	19.6
Monitoring	Laboratory test, e.g. HbA1C	103	12.4
	Non-laboratory follow-up, e.g. BP	51	6.1
	Both laboratory and non-laboratory monitoring	7	0.8
	Subtotal	61	19.3
Lifestyle recommendation	Smoking cessation	17	2.0
	Diabetes management	11	1.3
	Diet or weight management	10	1.2
	Multiple recommendations	10	1.2
	Exercise	9	1.1
	Other lifestyle recommendations	6	0.7
	Mental health related, e.g. stress reduction	5	0.6
	Sleep related	3	0.4
	Subtotal	71	8.5
Total		833	–

*BP* blood pressure, *DAA* dose administration aid, *HbA1c* glycated haemoglobin

^a^Non-prescription medication included complementary and alternative medications, and bush medicine

**Table 5:** Pharmacists’ recommendations (*n* = 278) for 170 MRPs documented in *PharMIbridge* RCT

Recommendations/actions		*n*	%
Change in pharmacotherapy	Dose increase	59	21.2
	Dose decrease	32	11.5
	Drug change	21	7.6
	Drug formulation change	4	1.4
	Dose frequency/schedule change	19	6.8
	Other changes to therapy	18	6.5
	Subtotal	153	55.0
Referral to other services	Refer to prescriber	67	24.1
	Refer for medication review	7	2.5
	Other referral required	3	1.1
	Subtotal	77	27.7
Information provision	Education or counselling session	27	9.7
	Written summary of medications	1	0.4
	Recommend dose administration aid	4	1.4
	Other written information	1	0.4
	Subtotal	33	11.9
Monitoring	Monitoring: laboratory test	7	2.5
	Monitoring: non-laboratory	5	1.8
	Subtotal	12	4.3
None	No recommendation necessary	3	1.1
	Subtotal	3	1.1
Total		278	–

## Discussion

The current study adds to the existing body of literature examining MRPs identified by pharmacists during medication reviews by presenting a parallel case study of two vulnerable and underserved populations. This work describes the numbers, types, and severity of MRPs identified in these populations using an established Australian classification system, as well as the recommendations made by pharmacists to address these MRPs. Adherence issues, undertreatment, and toxicity and adverse reactions were commonly identified in both populations, demonstrating priority areas to be addressed in these populations.

The DOCUMENT classification system was used to classify MRPs by *IMeRSe* and *PharMIbridge* pharmacists, enabling descriptive cross-comparison of the MRPs reported in two at-risk populations. ‘Compliance’ was the most and second-most common MRP identified during the medication reviews with *IMeRSe* and *PharMIbridge* consumer participants, respectively. Although there is insufficient evidence to demonstrate whether there is a significant difference in medication adherence between Indigenous Australians and the general population, healthcare professionals believe adherence is a significant issue for Indigenous Australians [38]. Furthermore, a recent review noted a lack of empirical evidence regarding medication-taking behaviours among Indigenous Australians [39]. Further work is required to explore contributing factors to adherence issues in this population, however, these may include previously identified challenges such as misconceptions regarding medications and lack of culturally appropriate information sources [20]. Barriers to accessing care have been acknowledged by policymakers, with system level strategies implemented to improve access to medicines and healthcare for Indigenous Australians [40]. Conversely, adherence issues with psychotropic medications is well documented in people living with SPMI, with many factors, including the adverse effects of psychotropic medications such as those reported as MRPs in this current study, contributing to poor adherence [41]. Moreover, polypharmacy is known to contribute to adherence issues; both population samples were taking a median of more than 5 medications (a commonly used definition for polypharmacy [42]), which may have influenced pharmacists flagging adherence as a potential issue.

There were also differences in MRPs between the two populations. For example, ‘Undertreated’ was the second-most common MRP identified in *IMeRSe*, reported twice as often as in *PharMIbridge*. This is consistent with the findings of a study conducted in remote Australia which found that 12% of elderly Indigenous participants were potentially under-prescribed medications [19]. This further emphasizes the continuing inequity in healthcare provision for Indigenous Australians, particularly those living in rural and remote areas [43, 44]. The least common MRPs identified in the *IMeRSe* sample, ‘Toxicity or adverse reaction’, was conversely the most common category in *PharMIbridge*, making up almost a quarter of all MRPs. This is not unexpected, given the significant adverse effect profile of medications used to manage SPMI and the incidence of high-dose psychotropics and psychotropic polypharmacy in this cohort [24, 45]. This finding further highlights the need for screening for, and managing, MRPs in this population. The inclusion of the M3Q tool [34], which is readily available for pharmacists to access [46], in the *Initial Health Review* may have facilitated further discussion with consumer participants about medication related adverse effects compared to other types of MRPs. Regardless, the identification of numerous MRPs, irrespective of type, across both populations indicates the importance of timely access to services which are tailored to the needs of individuals.

When comparing the collective study results to the PROMISe trials, which piloted, validated, and refined the DOCUMENT system in a general population, differences can be seen in the nature of the MRPs reported. In PROMISe, ‘Drug selection’ and ‘Education or information’ were the two most common categories identified (30.7% and 23.7%, respectively) out of 5948 identified MRPs [35]. This differs to the most common categories identified in *IMeRSe* and *PharMIbridge*, namely ‘Compliance’, ‘Undertreated’, and ‘Toxicity or adverse reaction’. This could be due to differences in trial populations, the interventions trialed, or the intended purpose. The DOCUMENT system was primarily used in the PROMISe trials to document clinical interventions made in response to dispensed prescriptions [35, 47], rather than being used to code MRPs identified during a medication review as used in *IMeRSe* and *PharMIbridge*. A study by Wang et al. which used DOCUMENT to classify MRPs identified during medication reviews with elderly ambulatory patients in China found ‘Drug selection’ and ‘Undertreated’ made up almost half of 1,188 MRPs [48]. This highlights that different populations may have specific needs and priorites and further work exploring these nuances could produce findings that are useful for clinicians and policymakers.

Most MRPs were rated as having a severity of either ‘Mild’ or ‘Moderate’ (Additional file 1: Supplementary table i), together making up 61.9% and 80.6% of severity ratings in *IMeRSe* and *PharMIbridge*, respectively. This indicates that pharmacists identified MRPs that, when addressed, would have improved compliance, or alleviated a minor symptom, or prevented a doctor visit. Wang et al*.* similarly classified 86% of MRPs identified among elderly ambulatory patients in these categories [48]. However, pharmacists in *IMeRSe* rated more than a quarter of MRPs as ‘High’ severity, indicating that they believed addressing the MRP prevented a hospital visit or referred participants to hospital. These findings highlight that identified MRPs could have potentially had a range of clinical outcomes if not identified and that there is room for new opportunities in primary care to identify and manage MRPs in these populations, to minimize serious sequelae.

In addition to examining the types and severity of MRPs, it is necessary to consider the implementation and use of the DOCUMENT classification system itself. During the validation process of DOCUMENT, a “moderate” level of agreement was found between raters, indicating that there was some inconsistency in classification of MRPs [35]. Similarly, differences between raters have been found in the classification of MRPs identified in medication reviews in a Norwegian study using a different classification system [49]. The potential for disagreement or misclassification must be considered when interpreting the results of the current study as independent coding and review of the assigned categories was not performed except for severity. Similar to *IMeRSe*, severity ratings made by pharmacists in the PROMISe trials were potentially over-stated [35]. However, the differences in severity ratings between the study pharmacists and the independent coder in the current study could possibly be attributed to various factors, including the information available to each at the time of coding, familiarity with the consumer and their medical and social history, and overall experience in conducting medication reviews and considering potential clinical significance of MRPs. Further training and consideration of pharmacists’ understanding of the clinical significance of MRPs should be considered. The quality of documentation of MRPs must be considered, not only to ensure accurate and clear clinical notes, but to also ensure meaningful communication regarding MRPs with other healthcare professionals, such as the prescriber. This is particularly important as referral to another professional or service was commonly recommended by pharmacists. It is essential to ensure that pharmacists are trained in clear and accurate clinical documentation.

### Strengths and limitations

The current study is strengthened by classifying the identified MRPs using an established and validated classification system designed for the Australian practice setting. Key strengths of both the *IMeRSe* and *PharMIbridge* interventions included ongoing follow-ups, which may facilitate the working relationship between consumers and community pharmacists. Although the two studies are heterogenous in design and direct comparisons between the two populations cannot be made, this study highlights the needs of the two underserved and vulnerable populations which may inform the implementation of targeted training and/or services. Furthermore, eligibility criteria for consumer participants in both studies included being at-risk of MRPs; this may have resulted in increased rates of MRPs identified in these two samples. It is unknown how generalizable these two samples are to the broader populations of Indigenous Australians and people living with SPMI. The physical health focus of the *PharMIbridge* intervention, may have resulted in comparatively less MRPs being identified out of all the issues due to the focus of training and overall design.

It is likely that trial pharmacists had varying degrees of experience conducting medication reviews and using the DOCUMENT classification system; this may have impacted how pharmacists conducted the reviews and classified MRPs. Some pharmacists had undergone additional training to become accredited pharmacists (i.e. able to conduct Home Medicines Reviews) which may also have impacted how they conducted the medication reviews in the two studies. It is also important to acknowledge that the independent coding was conducted by a single accredited pharmacist and a panel approach to classification may have yielded different results. Lastly, outcomes for individual MRPs, such as GP acceptance rates, further healthcare use due to MRPs, and resolution of MRPs, were unable to be tracked.

## Conclusion

Community pharmacists identified a range of MRPs with varying levels of severity experienced by two at-risk and underserved populations, namely Indigenous Australians and people living with SPMI, when conducting novel interventions which included medication review. Non-adherence to medication and the presence of toxicity or adverse reactions were among the most reported MRPs. Pharmacists proposed a variety of strategies to address the MRPs, most often involving a proposed change to pharmacotherapy. These findings highlight specific needs of the two populations and emphasize that there is room for more targeted approaches to identifying and managing MRPs in primary care and tailored community pharmacist-led interventions may be of value in this space.

### Supplementary Information

**Additional file 1: Table S1**. Severity descriptors as used in the PROMISe trials. **Table S2**. Examples of MRPs documented by pharmacists using the DOCUMENT framework. **Table S3**. Examples of MRPs documented by pharmacists for each of the severity classifications.

## Data Availability

The datasets generated and analysed for this study are not publicly available as consent from participants was not sought to share the data more widely than for the purpose of this study. Data are not publicly available as consent for data sharing was not obtained from participants.

## Footnotes

1 Please note that the use of the term ‘Indigenous’ in this paper includes all Aboriginal and Torres Strait Islander people and acknowledges their rich traditions and heterogenous cultures.

2 It is acknowledged that Aboriginal and Torres Strait Islander people may also live with SPMI and the authors are not suggesting these are two discrete populations. Some participants in *IMeRSe* lived with SPMI and some participants in *PharMIbridge* identified as Aboriginal or Torres Strait Islander.
