# Supplementary material for: Medication-related problems identified by community pharmacists: a descriptive case study of two Australian populations
Source: J Pharm Policy Pract. 2023 Nov 2;16:133. doi: 10.1186/s40545-023-00637-x (PMC10621197; doi:10.1186/s40545-023-00637-x)
Supplement: Supplementary file 1 — Additional file 1: Table S1. Severity descriptors as used in the PROMISe trials. Table S2. Examples of MRPs documented by pharmacists using the DOCUMENT framework. Table S3. Examples of MRPs documented by pharmacists for each of the severity classifications. [file 40545_2023_637_MOESM1_ESM.pdf]

Medication-related problems identified by community pharmacists: a descriptive case study of two populations

Additional file 1: Tables

Table S1: Severity descriptors as used in the PROMISe trials (1)

Severity level	Descriptor
High (S4)	When, if the intervention did not occur, it was likely that the patient would have had to go to a hospital because of the consequences. Also covers the situation where the pharmacist needs to refer the patient to a hospital because of the seriousness of the situation
	When, if the intervention did not occur, it was likely the patient would have required assistance from a regular nurse visit, or would have required placement into residential care
Moderate (S3)	When, if the intervention did not occur, it was likely that the patient would have had to visit the doctor because of the consequences. Also covers the situation where the pharmacist needs to refer the patient to the doctor because of the seriousness of the situation
Mild (S2)	Consequences to the patient are that they have improved compliance or improved or prevented a minor symptom. The sign or symptom should not require a doctor's visit to treat
Low (S1)	Consequences to the patient are related to costs or information only
Nil (S0)	No consequence to the patient

Table S2: Examples of MRPs documented by pharmacists using the DOCUMENT framework (1)

MRP category	IMeRSe example	*PharMIbridge* example
**D**rug selection	"Need for aspirin and whether it may be contributing to her reflux problem"	"Whether quetiapine is still the best medication for bipolar diagnosis"
**O**ver or underdose	"Quetiapine 200 mg was making him drowsy and he felt that he was eating more and gaining weight from it ... he's biting to half the tablet at night"	"Patient is experiencing diarrhea multiple times a day … still taking [docusate and sennosides] 2 times a day"
**C**ompliance	"[Blood pressure] 168/95 [mmHg] and hadn't taken pills this morning"	"There are days where [consumer participant] does not feel like taking his medications … discards them down the toilet"
**U**ndertreated	"Patient's blood pressure was checked and was moderately high, averaging 150/82 [mmHg] over 3 readings"	"Current meds and previous medications don't work as well as they could for his mood"
**M**onitoring	"Flupentixol monitoring of [cardiovascular disease] … consider checking [electrocardiogram] and monitor lipids and glucose"	"Monitoring of B12 as pantoprazole and metformin may cause malabsorption of B12"
**E**ducation or information	"[Consumer participant] was unclear about her medications and what each one does"	"Worries about lithium blood tests as she doesn't want anything to go wrong with them"
**N**ot classifiable	"Takes 1 x aspirin 100 mg each night, but i [night-time] aspirin is not on [consumer participant's] current medication summary"	"Still experiences back pain and takes [cocaine] to help"
**T**oxicity or adverse reaction	"Currently experiencing a dry cough while taking ramipril"	"Clozapine causing excessive drowsiness"

Table S3: Examples of MRPs documented by pharmacists for each of the severity classifications

**Severity**	***IMeRSe* example**	***PharMIbridge* example**
High	"[Participant's blood pressure] was 92/60 [mmHg] and pulse 43 and it had been consistently low over the past few weeks but not as low as today… He has previously had a fall when his [blood pressure] was low"	"There is an ongoing issue regarding the prescribing of [participant's] alprazolam. His specialist only gives him 30 days' worth with no repeat. Sometimes [participant] will run out on a weekend and experiences benzodiazepine withdrawal. This cycle occurs every month and causes significant anxiety for [participant]"
Moderate	"[Sub]optimal heart failure treatment"	"Lethargic, drowsy & " foggy " in the morning after taking medications"
Mild	"[Participant] reports frequent nausea, and is currently taking 2 x 1 g immediate release metformin tablets each morning (when standard dosing of immediate release metformin is max 1g at any one dose and divided doses (e.g., [twice a day]) if exceeding this)"	"Medication causing weight gain"
Low	"Possible unintentional [non]compliance without [dose administration aid]"	"Remembering morning meds"
Nil	"Relies on natural medicines to control her pain, states that the gumbi gumbi is very effective for fibromyalgia"	"[Participant] is interested in trialling a does reduction of mirtazapine"

Reference

(1) Williams M, Peterson GM, Tenni PC, Bindoff IK, Stafford AC. DOCUMENT: A system for classifying drug-related problems in community pharmacy. Int J Clin Pharm. 2012;34(1):43-52. doi: 10.1007/s11096-011-9583-1.
